# Crystallographic education in the 21st century

**DOI:** 10.1107/S1600576715016830

**Published:** 2015-10-13

**Authors:** Saulius Gražulis, Amy Alexis Sarjeant, Peter Moeck, Jennifer Stone-Sundberg, Trevor J. Snyder, Werner Kaminsky, Allen G. Oliver, Charlotte L. Stern, Louise N. Dawe, Denis A. Rychkov, Evgeniy A. Losev, Elena V. Boldyreva, Joseph M. Tanski, Joel Bernstein, Wael M. Rabeh, Katherine A. Kantardjieff

**Affiliations:** a Vilnius University Institute of Biotechnology, Graiciuno 8, LT-02241 Vilnius, Lithuania; b Department of Chemistry, Northwestern University, 2145 Sheridan Road, Evanston, Illinois 60208, USA; c Nano-Crystallography Group, Department of Physics, Portland State University, PO Box 751, Portland, OR 97207-0751, USA; d Crystal Solutions, LLC, Portland, OR 97205, USA; e 3D Systems Corporation, Wilsonville, SW Parkway Avenue 60 E – 61, Wilsonville, OR 26600, USA; f Department of Chemistry, University of Washington at Seattle, Box 351700, Seattle, WA 98195, USA; g Molecular Structure Facility, Department of Chemistry and Biochemistry, University of Notre Dame, Notre Dame, IN 46556-5670, USA; h Department of Chemistry and Biochemistry, Wilfrid Laurier University, Waterloo, ON, Canada; i Institute of Solid State Chemistry and Mechanochemistry, Kutateladze 18, Novosibirsk 630128, Russian Federation; j REC-008, Novosibirsk State University, Pirogova 2, Novosibirsk 630090, Russian Federation; k Department of Chemistry, Vassar College, 124 Raymond Avenue, Poughkeepsie, NY 12604, USA; l New York University Abu Dhabi, PO Box 129188, Saadiyat Island, Abu Dhabi, United Arab Emirates; m College of Science and Mathematics, California State University, San Marcos, Craven 6211, USA

**Keywords:** teaching, education

## Abstract

There are many methods that can be used to incorporate concepts of crystallography into the learning experiences of students, whether they are in elementary school, at university or part of the public at large. It is not always critical that those who teach crystallography have immediate access to diffraction equipment to be able to introduce the concepts of symmetry, packing or molecular structure in an age- and audience-appropriate manner. Crystallography can be used as a tool for teaching general chemistry concepts as well as general research techniques without ever having a student determine a crystal structure. Thus, methods for younger students to perform crystal growth experiments of simple inorganic salts, organic compounds and even metals are presented. For settings where crystallographic instrumentation is accessible (proximally or remotely), students can be involved in all steps of the process, from crystal growth, to data collection, through structure solution and refinement, to final publication. Several approaches based on the presentations in the MS92 Microsymposium at the IUCr 23rd Congress and General Assembly are reported. The topics cover methods for introducing crystallography to undergraduate students as part of a core chemistry curriculum; a successful short-course workshop intended to bootstrap researchers who rely on crystallography for their work; and efforts to bring crystallography to secondary school children and non-science majors. In addition to these workshops, demonstrations and long-format courses, open-format crystallographic databases and three-dimensional printed models as tools that can be used to excite target audiences and inspire them to pursue a deeper understanding of crystallography are described.

## Abstract 2

Methods and outcomes for teaching crystallography in graduate, post-graduate and secondary school environments are presented. This is an extended report based on the ideas presented in the MS92 Microsymposium at the IUCr 23rd Congress and General Assembly in Montreal.

## 1. Introduction   

More than 100 years have passed since the remarkable discovery of X-rays (Röntgen, 1895 ▸, 1896 ▸) and the seminal experiments of Friedrich, Knipping and Laue (Walther *et al.*, 1912 ▸). As a scientific discipline, crystallography has contributed many ground-breaking achievements in elucidating the structure of matter at or near the atomic level of detail, from the simplest inorganic salts to functional complexes or proteins, resulting in 29 Nobel Prizes (IUCr, 2014 ▸). In the wake of 2014, the International Year of Crystallography (IYCr2014; http://www.iycr2014.org/), the highly interdisciplinary nature of crystallography and its diverse community of practitioners and users offers opportunities to invigorate interest in STEM (science, technology, engineering, mathematics) as well as providing a vehicle for demonstrating the importance of validation, critical analysis and hypothesis testing.

Crystallography can be introduced in secondary school and undergraduate chemistry courses through crystal growth experiments and crystal growing competitions. The aesthetic qualities of nicely shaped crystals can motivate students to further pursue experimental and theoretical studies of crystallography; at the same time they permit educators to introduce various concepts of crystallography and demonstrate a wide variety of chemical and physical processes that take place during crystal growth. Such practical and theoretical courses can also serve as a good preparation for further studies using X-ray diffraction techniques.

Although many undergraduate science educators have recognized X-ray crystallography as an important scientific practice, the topic is not universally covered with any great depth in modern curricula, or even introduced. In 1988, Rossi & Berman (1988 ▸) noted that, while it is important to teach the fundamentals of crystallography to beginning science students, the study of crystallography has largely been neglected. At that time, ready access to diffraction instrumentation was sporadic and certainly not common in the undergraduate environment. In the United States in particular, crystallography was often found practiced in university chemistry departments, whose degree programs are typically certified by the American Chemical Society (ACS). With no formal requirement to purchase such expensive instrumentation in a certified program, departments were not compelled to obtain diffractometers (Pett, 2010 ▸; ACS, 2008 ▸). Ten years later, diffractometers became more robust and readily available, even in the undergraduate environment. Predominantly undergraduate institutions in particular embraced the pedagogy of enabling undergraduates to conduct hands-on research in crystallography (Crundwell *et al.*, 1999 ▸). Today, CCD detectors provide for easy and fast data collection, even by remote access, and modestly priced powder diffraction and single-crystal desktop instruments have afforded the opportunity to bring X-ray diffraction into undergraduate teaching laboratories. However, they are still not required instruments in ACS certified chemistry departments (ACS, 2014 ▸).

Despite these efforts to bring crystallographic science into the classroom and the teaching laboratory, widespread incorporation of crystallography and diffraction methods into core curricula remained elusive. Then, in 2006, the American Crystallographic Association (ACA) and The United States National Committee for Crystallography produced a crystallography education policies document (ACS, 2014 ▸) which, for the first time, articulated guidelines to professional societies, academic departments and funding agencies for crafting future crystallography curricula that adequately address the needs of the entire scientific community. This began more aggressive efforts on the part of the ACA and the IUCr Education Committee and Commission on Crystallographic Teaching to regularly organize microsymposia focused on incorporating, in pedagogically sound ways, crystallographic science, both methods and content, into core undergraduate science curricula. The ACA and IUCr web sites hold many materials described in these symposia, and there are a number of excellent examples of exercises and content designed to teach students about diffraction and the valuable contributions of crystallography to modern science and technology (Ortiz *et al.*, 2013 ▸; Wilson *et al.*, 2012 ▸; Pett, 2010 ▸; Battle *et al.*, 2010 ▸; Faust *et al.*, 2008 ▸, 2010 ▸; Kantardjieff, Lind *et al.*, 2010 ▸; Kantardjieff, 2010 ▸).

In 2010, *Journal of Applied Crystallography* dedicated a special volume to a review of crystallography education and training progress, the potential of Web 3.0 technologies, and the challenges facing the crystallographic community (Kan­tardjieff, Kaysser-Pyzalla & Spadon, 2010 ▸). Like the objectives of that special volume, the microsymposia sponsored by the Commission on Crystallographic Teaching at the recent IUCr Congress and General Assembly in Montreal aimed to provide an international forum for disseminating proven pedagogies, superior curriculum materials, innovations in teaching crystallography and successful education outreach efforts. This article describes the content from the second of two microsymposia on crystallography education and training at the 2014 IUCr Congress and General Assembly in Montreal. Talks by Peter Moeck, Joseph Tanski, Wael Rabeh and Louise Dawe described innovative ways to incorporate crystallography into undergraduate college curricula using crystal growth activities, advanced laboratory experiments, critical analysis of the structural literature and three-dimensional printed models. Elena Boldyreva presented activities in Russia that successfully incorporate crystallography topics into secondary school curricula. Gabriela Diaz De Delgado gave an historical account of crystallography education and training in Latin America. Although the coverage of the microsymposia and of the present article by no means exhausts all possible ways to teach crystallography owing to time and space constraints, we hope that it will provide a representative coverage of some of the most fruitful ideas, events and topics that are relevant in the new century of crystallographic education, and the provided references will serve as further guidance. The crystallographic community must continue to advance crystallography education and training if our science is to remain vibrant in the 21st century. The International Year of Crystallography has provided renewed inspiration with these presentations.

## 2. Teaching crystallography to undergraduates   

### 2.1. Teaching crystallography through activity-based learning   

Undergraduate course descriptions in areas of general, organic and inorganic chemistry and spectroscopy often include topics such as trends in the periodic table, chemical bonding, three-dimensional structure of molecules, stoichiometry, introduction to reactions and reactivity (*e.g.* CH110: *Fundamentals of Chemistry I*, Wilfrid Laurier University, Waterloo, Ontario, Canada^1^); molecular symmetry, introduction to molecular orbital theory, structure and stereochemistry of typical inorganic compounds (CH225: *Inorganic Chemistry I* 1); and solving structural and stereochemical problems in organic chemistry (CH303: *Spectroscopic Methods in Organic Structure Elucidation: Inorganic Chemistry I* 1). These des­criptions are clearly amenable to the study of X-ray crystallography and the incorporation of structural studies into course learning outcomes. Two questions then arise: how can this be done at the undergraduate level, where resources and student background knowledge are often limited; and how can the maximum number of students be engaged in these learning activities? Answers can be found partly in the pedagogical approach and in careful construction of learning activities.

A constructivist framework for teaching and learning at the undergraduate level provides the space for students to make connections with their environment, including their social environment, and to develop mastery of course learning outcomes through challenge and puzzlement (Savery & Duffy, 1999 ▸; Jonassen & Rohrer-Murphy, 1999 ▸). The example under consideration here is how students in a third-year undergraduate course, CH390: *Chemical Literature and Scientific Communication*, at Wilfrid Laurier University during the Winter 2013 term learned about elements of X-ray crystallography, and then acted as peer instructors to teach concepts of crystallography to first-year general chemistry students. In so doing, students in both courses engaged in active learning (Freeman *et al.*, 2014 ▸) and reflection, whereby they uncovered connections between their lives and the subject content they had studied.

The course outcomes for CH390 (see Appendix 1 in the supporting information) were based on the recommendations from the Special Libraries Association, Chemistry Division, and the American Chemical Society, Division of Chemical Information (2011 ▸). Specific focus was placed on those outcomes listed in Table 1 ▸.

The course projects were then designed on scaffolding principles, with the eventual transfer of responsibility from experts (course instructors) to self (which requires metacognition on the part of the student) and finally to reciprocal (peer) scaffolders. Holton & Clarke (2006 ▸) define scaffolding as an act of teaching that (i) supports the immediate construction of knowledge by the learner and (ii) provides the basis for the future independent learning of the individual. As in construction, project scaffolding enables learners to reach otherwise unattainable outcomes. Its methodologies facilitate mastery of new concepts while challenging and correcting misconceptions. Finally, scaffolding is not seen in the final product; while the learner may be aware of the process, once an outcome is achieved, the method by which this was accomplished may not be obvious (Lajoie, 2005 ▸). The roles of instructors and students can be defined in project scaffolding but are not restricted to traditional contexts. In fact, project scaffolding lends itself well to flipped classroom approaches, where students are empowered to guide their own learning.

**Figure 1:**
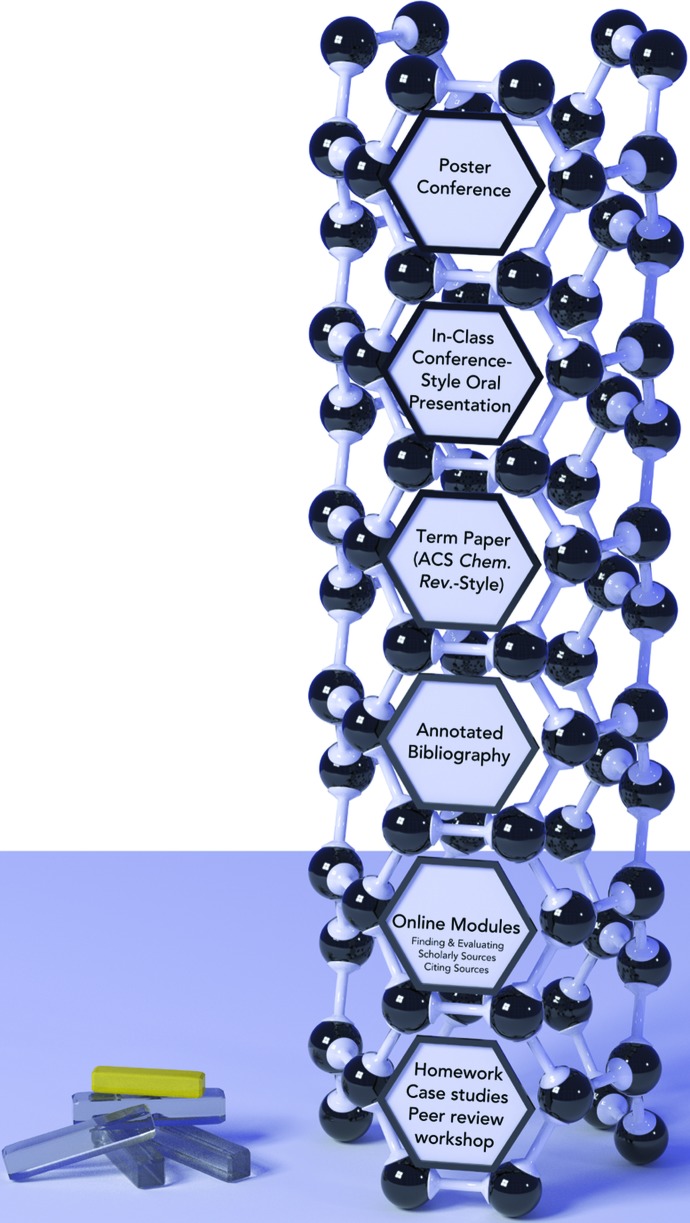
Project scaffolding framework for CH390: *Chemical Literature and Scientific Communication*.

The method by which this was achieved in CH390 first required students to select a topic from a provided list (see Appendix 2 in the supporting information for the list of topics and accompanying instructions). Students sent their top three selections, in addition to a comment on what they enjoyed about chemistry, to the course instructor. As it was unlikely that many students had prior experience with these concepts, it was important to match their interests with an appropriate project topic in order to increase the likelihood that the students would find meaning in their work. The first course projects, which formed the base of the course scaffold (Fig. 1 ▸), were developed by Wilfrid Laurier University’s Study Skills and Supplementary Instruction Center and required students to complete four online assignments: Finding Scholarly Sources, Evaluation Sources, Citing Sources and Creating an Annotated Bibliography. The assignments complemented in-class content, and students received graded feedback to guide their work into the next stage of evaluation. For each of these modules, students were required to follow ACS style guidelines in order to fulfill the course outcomes and their research had to be focused on their assigned topic. These preliminary assignments started their transition to self-scaffolders.

Students then worked through the major assigned course projects: preparing an annotated bibliography, writing an ACS *Chemical Reviews* style term paper, giving an in-class oral presentation and presenting at a public poster conference. At each stage course outcomes were supported by expert instruction – for example, university librarians taught about creating an online presence, open-access publishing and research data management – but all crystallographic content was obtained through the student’s own research. The final course project was a public poster display in which the students had to communicate their crystallography knowledge to a diverse public audience made up of non-experts, flipping the classroom entirely so that students taught the course instructors and other undergraduates about their assigned topics. In order to ensure a large turnout at this event (295 first-year students and other members of the community), and to facilitate chemistry education beyond the textbook content often associated with first-year chemistry, the students who were registered in CH111: *Fundamentals of Chemistry II* at Wilfrid Laurier University during the Winter 2013 term were assigned to attend this poster presentation and to write a short summary of what they learned from one poster and why they found that topic interesting. Student responses were revealing, with many relating posters to their own personal conditions or concerns about the environment and beyond. These insights provide avenues for making future connections between regular course content and the social environment in which students live and learn.

The scaffolded approach to learning used in CH390 enabled student transformation through the role of metacognition in connecting content knowledge with research and communication skills, whereby students learned skills towards self-scaffolding and developed connections to the various learning resources on campus, such as the Writing Center, Library and Online Research Supplemental Instruction, as these groups facilitated learning by acting as expert scaffolders. Students practiced transdisciplinary inquiry as, although course outcomes related to research, writing and communication, subject learning goals related to the broad field of crystallography. Students found broad meaning in their research, in part because they were first surveyed, and then individually interviewed, to match their chemistry interests to crystallographic research topics. Finally, the culmination of CH390 in a peer-shared poster conference where students acted as reciprocal scaffolders by teaching attendees the concepts they had only recently mastered facilitated a sense of community between students, experts and a larger audience.

### 2.2. Crystal growth as a hands-on method to teach diverse scientific concepts   

At New York University Abu Dhabi (NYUAD), a crystal growth laboratory engages students with diverse scientific backgrounds in learning how to grow and handle crystals. This research-based learning environment with its hands-on experience builds scientific knowledge, as well as laboratory and project management skills. *Foundations of Science*, a unique introductory science course taken by first-year students majoring in science and engineering disciplines, integrates basic concepts from biology, chemistry and physics in an innovative science course. The crystal growth experiment is included in the laboratory component of the course. Additionally, the *Domain of Crystals*, a course that is part of the core curriculum for non-science students, makes use of the crystal growth laboratory to introduce concepts from the natural world, and develops laboratory and project management skills for non-science majors. Overall, the crystal growth laboratory is designed to accommodate students with different scientific backgrounds.

**Figure 2:**
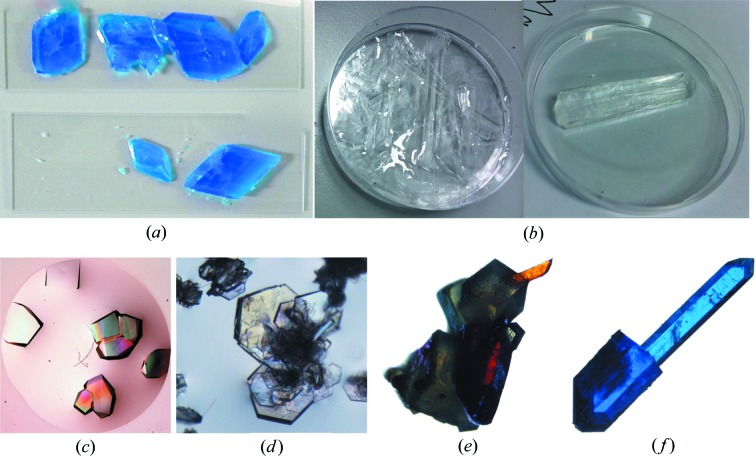
Pictures of crystals from the *Foundation of Science* laboratory course at New York University Abu Dhabi. (*a*) Crystals of copper(II) sulfate pentahydrate grown at different temperatures using the evaporation method. (*b*) Crystals of zinc sulfate grown with or without a magnetic field using the evaporation method. (*c*), (*d*) Micro-sized crystals of lysozyme and cysteine grown *via* the hanging-drop method using Crystal Screen from Hampton Research. (*e*) Micro-sized crystals of a mixture of potassium ferrocyanide, K
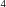
Fe(CN)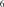, and potassium chloride. (*f*) Micro-sized crystals of a mixture of calcium acetate, copper(II) acetate and copper chloride. The detailed setup of the crystal growth experiments is presented in Appendix 3 of the supporting information.

The original inspiration for the laboratory came from the classic book *Crystals and Crystal Growing* by Holden & Singer (1960 ▸). However, the laboratory was expanded from having an emphasis on crystallizing simple inorganic salts to including the crystallization of macromolecules in a research-driven environment. The laboratory starts with a crystal growth project where students initiate a more comprehensive plan to grow crystals of small inorganic molecules and macromolecules. Similar to projects carried out in more advanced courses, students write an outline of their projects. This is further developed into a full proposal through a literature search that is stimulated by in-class discussions and peer review. Students choose the theme of their project and develop their ideas into laboratory protocols through additional literature searches and consultation with the laboratory instructor. While still evolving, the laboratory has seen a variety of mainly student-initiated investigations that not only explore a variety of methods to grow crystals but also examine the effect of temperature (Fig. 2 ▸ *a*), magnetic field (Fig. 2 ▸ *b*), vibration and pH on crystal growth. The students’ projects, which were as diverse as their backgrounds, included growth of micro-sized crystals of lysozyme (Fig. 2 ▸ *c*) and cysteine (Fig. 2 ▸ *d*); crystals of a mixture of potassium ferrocyanide and potassium chloride (Fig. 2 ▸ *e*); and others including a mixture of calcium acetate, copper(II) acetate and copper chloride (Fig. 2 ▸ *f*).

While one objective of the laboratory is to learn how to grow and handle crystals, the exercise fosters the acquisition of basic scientific knowledge and hands-on experience and develops essential intellectual skills. In the crystal laboratory project, students are evaluated at different stages of their projects. A two-page outline is due at the beginning of the project. Students will take comments from their instructor and further develop the outline into a full proposal that includes methodology and a detailed plan to carry out their project. The proposal will incorporate the ideas and methods needed to carry out the project. The instructor will give feedback after the proposal is peer reviewed by other students in the course, and the instructor will return the proposal prior to students starting the project. Upon completing the experiments and collecting all the data needed to draft a full report including a results and discussion section, the students will submit their reports to the Crystal Growth Symposium, an end of semester student conference. Students will present their projects as a short two minute oral presentation or a short video at the beginning of the symposium depending on the number of student groups, followed by a poster presentation session. In the symposium, students practice their presentation skills and learn how to present their results and project in a scientific setting. Different academic judges including the laboratory instructors evaluate the students’ presentations and overall projects. Trophies and prizes are also presented to the top three groups as a means to encourage the students participating in the symposium.

### 2.3. Integrating chemical crystallography into undergraduate teaching laboratories   

In cases where diffraction equipment is available either in-house or nearby, it is advantageous to incorporate use of the diffractometer into undergraduate coursework. An example, outlined here, is a discovery-based spectroscopy and X-ray crystallography laboratory module designed for undergraduate students in a small to medium size advanced teaching laboratory (Aldeborgh *et al.*, 2014 ▸). This teaching module serves to integrate chemical crystallography into the laboratory teaching curriculum when an X-ray diffractometer is available.

In this discovery-based module, each student is given a unique unknown solid organic compound to determine its identity by ^1^H, ^13^C and distortionless enhancement by polarization transfer (DEPT) nuclear magnetic resonance (NMR) spectroscopy, IR spectroscopy, and gas chromatography/mass spectrometry (GC/MS). The small-molecule organic compounds are selected in part because their crystal structures have not been published. Students may then have the opportunity to be part of the writing up and publication of the results of their work, integrating teaching with discovery-based research and the communication of new scientific knowledge.

For the spectroscopic analysis, students are guided through hands-on data collection on each instrument, and lectures and workshops are given to provide some background on how the instruments function, the theory behind each technique and the interpretation of various spectra. The coupling of information about the CH(*X*) framework and functional groups from NMR data, functional group information from IR data, and mass, fragmentation and even isotope information from GC/MS data should be sufficient for the students to propose the identity of their small organic molecule. After completing a laboratory report detailing the identity of the organic compound, the students then perform recrystallizations and determine theX-ray crystal structure of the compound. Before introducing the students to the diffractometer, it is advantageous for them to carry out simple recrystallizations, such as slow evaporation from several different solvents, in order to reiterate that the best possible quality crystals should be sought for the diffraction experiment. As with the spectroscopic techniques, each student is then guided in selecting a crystal with a polarized light microscope, mounting the sample, collecting the data, and solving and refining the crystal structure. Further, assigning guided reading of crystal structure reports in *Acta Crystallographica* and other chemistry journals provides a clear expectation of the report required of the students: a draft in the format of an *Acta Crystallographica* structure report. The spectroscopy report and *Acta Crystallographica* draft afford two written assignments that can be graded and serve as an assessment of student performance.

In the written structure reports in particular, it is valuable to ask the students to critically analyze the packing diagram and analyze any inter- or intramolecular interactions. These interactions can include traditional hydrogen bonding, non-traditional weak C—H⋯*X* hydrogen-bonding interactions, π stacking, halogen–halogen interactions and other interactions such as *E*—H⋯π interactions. Free and easy to use programs such as *Mercury* (Macrae *et al.*, 2006 ▸) and *Olex2* (Dolomanov *et al.*, 2009 ▸) are valuable in analyzing the packing diagrams and intermolecular interactions. Hydrogen-bonding (Arunan *et al.*, 2011 ▸), weak C—H⋯*X* hydrogen-bonding (Desiraju & Steiner, 1999 ▸) and halogen–halogen interactions (Pedireddi *et al.*, 1994 ▸) and π stacking (Hunter & Sanders, 1990 ▸; Lueckheide *et al.*, 2013 ▸) are now well documented in the scientific literature.

The process of carrying out the actual experiment of a crystal structure determination with students enrolled in an advanced undergraduate teaching laboratory and requiring them to write up the results is a valuable and high impact way to expose undergraduates to crystallography, molecular structure, intermolecular interactions, scientific writing and the publication of X-ray structural results. Students express an interest in seeing their results published, which adds excitement to the course.

## 3. Extracurricular teaching opportunities for crystallography   

### 3.1. A crystallographic workshop to supplement core curricula   

What can be done when X-ray crystallography is a necessary component of research but formal courses are non-existent in a student’s home institution? In the United States, undergraduate chemistry curricula are accredited by the American Chemical Society, whose guidelines make no specific requirement for teaching X-ray crystallography (ACS, 2008 ▸). As such, and owing to the large expense of purchasing and maintaining single-crystal diffraction equipment, most undergraduate students have only a cursory experience with this technique. On the graduate level, some departments offer elective courses in crystallography, but it is rare to find a course that combines theoretical and practical education. With such a dearth of learning opportunities in traditional curricula, students whose research depends significantly on X-ray crystallography seek other educational resources. One such outlet which has proven highly successful is the American Crystallographic Association’s Summer Course in Chemical Crystallography.

The ACA Summer Course has been an institution for crystallographic education in the United States since 1992, though the inspiration for the course began back in 1973 under the guidance of Robert Sparks at Syntex (Byram, 2001 ▸; Stern *et al.*, 2014 ▸). This early course was well received by students, but unfortunately plans for future courses never came to fruition. It was not until nearly 20 years later that funding became available to support an annual summer course devoted to teaching crystallography.

In 1992 the course was held at the University of Pittsburgh owing to its proximity to that year’s ACA meeting and to other practical considerations such as equipment and instructor availability. In the intervening years, the course has had myriad instructors and has been held at the University of Georgia, Indiana University of Pennsylvania, and in its current incarnation jointly at The University of Notre Dame and Northwestern University. While several topics have been introduced (powder X-ray diffraction) and removed (macromolecular crystallography), the general curriculum and format of the ACA summer school has remained unchanged since its inception.

The summer school has long been seen as a resource for burgeoning crystallographers at different points in their careers and from locations around the world. Attendees include graduate and undergraduate students, post-docs, faculty members, and industry researchers. This variety of attendees, who bring with them their own unique questions and research samples, enriches the overall course experience. Because there are no requirements for past experience with crystallography, course topics must reach from basic principles and theory to advanced themes such as twinning, modulation and other novel techniques. Additionally, students with stronger backgrounds often become teachers to those who may be struggling with new concepts.

For regions that lack significant training in crystallography on the collegiate or graduate level, hosting a summer course can prove to be a viable option. As recent IYCr2014 Open Labs have shown (http://www.iycr2014.org/openlabs), there exists a need for crystallographic education throughout the world: specifically in developing nations. A successful course should be no shorter than one week and should include both theoretical and practical instruction. As such, it is important to host the school in a location where diffraction equipment is available, or where vendor sponsors can easily locate loaned instruments. While most attendees will probably bring their own laptop computers, it is advantageous to have a set of computers with all necessary software installed before the course begins. This ensures that each student will have the same environment and that all programs will be known to work without intervention. Additionally, having a repository of all test or demonstration data and freely available software in a shared location such as Dropbox will allow students to take these things with them and practice on their own after the course is over.

Faculty instructors ideally are volunteers who are willing to cover lectures for several topics and provide support for students, either individually or in groups, as they work up their own structures. Though it is often difficult to secure instructors for the length of the entire course, it is important to have enough experts on hand to answer questions that arise, specifically toward the end of the school when students are most likely to encounter troublesome refinements or difficult concepts. The ACA Summer Course has had the good fortune to maintain a very good student/teacher ratio, averaging about 2–3 students per instructor over the duration of the course.

Ultimately, once students complete the course, school or workshop, they should be able to bring most of their crystal structures to publication. However, it is often the case that course attendees require additional assistance. To this end, maintaining contacts with instructors and with other students serves to provide continuing support, strengthening and growing the entire crystallographic community.

### 3.2. Teaching in a secondary school   

Currently, most secondary school curricula provide a broad variety of compulsory courses giving a solid basis for furthering student progress at university. These fundamental courses are needed in order to successfully tackle more advanced courses and this demands hard work from every student. There is the belief that in this type of educational system the students lose the sense of adventure, discovery and research which is so important for children from a psychological point of view and which is extremely useful for developing future scientists. It becomes evident that the main question is whether compulsory subjects and open classrooms can be combined efficiently, in order to support students in their self-realization needs and provoke interest in mundane school subjects.

One of the most charming and breathtaking things in the world is creating something new. A simple opportunity of such a creative activity is provided by growing crystals. The ‘magic’ that occurs when a clear solution transforms into a perfect crystal can serve as a true bridge to the scientific world for the youngest researchers at a secondary school. Physical and chemical processes that occur during crystal growth can be explained using basic concepts of physics, chemistry, crystallography and mathematics, which provide the necessary framework for implementing this additional activity into the general secondary school curriculum. These concepts need not be explained in depth as on the university level; however, the main ideas and concepts can be introduced to younger students. Educators must find the proper balance between ‘strict’ on one side and ‘accessible’ or ‘interesting’ on the other. After the basics are understood and the techniques of crystal growth are successfully implemented for several compounds, students are ready to move further. Single-crystal and powder diffraction techniques can be shown, and even the principles of structure solution can be illustrated for the simplest cases.

There are many important issues that should be considered when one is organizing such hands-on experiments: safety, laboratory equipment, substances for crystallization *etc*. However, the most critical issue in the teaching process at a secondary school is finding appropriate teachers. It is important for the teacher to be able to explain all the processes as simply as possible, switching on imagination and training logical thinking of students of different ages, starting from the youngest children (6–7 years old). Moreover, it is important to combine practical work in the school laboratory with the explanation of the experiments during lectures. This combination of theory and practice is the key issue of the whole process of teaching crystallography at a secondary school.

It is extremely difficult to arrange all the required equipment in one location. Experience from over five years of teaching crystallography at a secondary school has shown that the best option is to arrange a cooperation between a university, a research institute(s) and a particular secondary school. In this example these are Novosibirsk State University, the Institute of Solid State Chemistry and Mechanochemistry Siberian Branch of the Russian Academy of Sciences, and School No. 162 of Novosibirsk, respectively. Undergraduates and graduate students are encouraged to work with school children, because the older students have the knowledge and experience of laboratory work while maintaining memories of their own learning experience. They can also establish a close contact with children, being closer in age. Novosibirsk University and the Institute of Solid State Chemistry give access to the necessary special equipment, such as state-of-the-art X-ray diffractometers, IR and Raman spectrometers, and many other instruments and accessories from a real scientific laboratory. All the formalities related to safety and other issues of organizing the process are arranged by the school staff.

The problems which the school children solve as their research mini-projects include growth of large perfect single crystals, crystallization of polymorphs of pharmaceuticals, producing co-crystals *versus* pure phases, modifying crystal habit by adding impurities and surfactants, studying the effect of crystallization conditions on the size, quality and mechanical properties of crystals, the role of substrates in crystallization, crystallization in gels, ‘crystal chemical gardens’ in relation to problems of biology (Kellermeier *et al.*, 2013 ▸; Cartwright *et al.*, 2002 ▸), and many others. Some examples were presented in contributions to the 27th European Crystallographic Meeting (ECM) in Bergen (Boldyreva, 2012 ▸; Losev *et al.*, 2012 ▸), ECM28 in Warwick (Losev *et al.*, 2013 ▸; Rychkov & Boldyreva, 2013 ▸) and the 23rd IUCr Congress in Montreal (Rychkov, Boldyreva *et al.*, 2014 ▸). The crystal growth techniques used have been described previously (Rychkov, Arkhipov & Boldyreva, 2014 ▸) and the general teaching strategy is close to the one used to teach crystallography to chemists at Novosibirsk State University (Boldyreva, 1993 ▸, 2010 ▸). Each student has to not only plan and perform a series of experiments, being assisted by a senior tutor, but record protocols for all experiments using a laboratory notebook as well as photographic and video recordings, and prepare a presentation and a poster describing the experiments, their results and interpretations. The students then present their work at a special School Session of a Student Research Conference of the Novosibirsk State University. In 2011 the best results were also presented by four school children in Grenoble, at the Institut Néel, at ESRF and at the Lycee ‘Champollion’. Remarkably, these children have since entered the Faculty of Natural Sciences of the Novosibirsk State University and are already in their third year studying chemistry.

**Figure 3:**
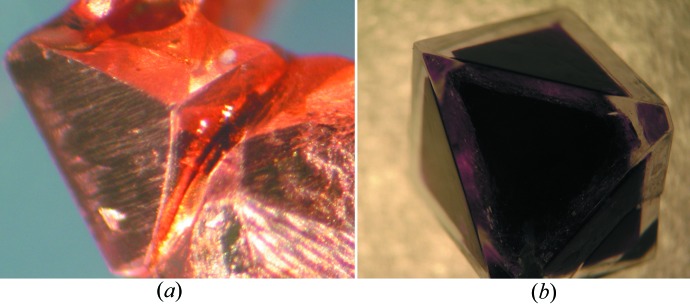
Examples of crystals grown by school children: (*a*) copper, (*b*) chromium potassium sulfate covered by aluminium potassium sulfate. See also http://iycr2014.org/participate/crystal-growing/entry?show=87794. A detailed description of the experimental setup to reproduce these crystals is provided in Appendix 4 of the supporting information.

It is important not to stop at the level of simple laboratory work, but to allow each student to plan and accomplish a research project, even if the project is a simple one and the results are obvious for the teacher, and to share with others all the difficulties of their research work, as well as the joys of success (Figs. 3 ▸–5 ▸ ▸). This educational course helps school children to develop experimental skills, to learn basic concepts in chemistry, physics and crystallography, and, no less importantly, overcome difficulties, become confident in their own abilities and experience the feeling of joy of examining the world. This model of ‘simulating the full path of a real scientific project’ helps children to understand what the job of a scientist truly is and helps us to recruit the most motivated children to the university later on. But even those children who will never become scientists themselves will remember this spirit of adventure combined with careful and persistent work in their future life. If they do so, the future society will be better educated and have a higher respect for science and knowledge, which is indeed the final aim.

## 4. Tools for teaching crystallography   

### 4.1. Use of open databases for crystallographic education   

Open crystallographic databases provide new opportunities for teaching students and involving them early in the curriculum in full-scale scientific research. For example, the Crystallography Open Database (COD; Gražulis *et al.*, 2009 ▸, 2012 ▸) and Protein Data Bank (PDB; Berman *et al.*, 2000 ▸) are used in the Vilnius University bioinformatics courses to provide students with an example of real-life data. While the pay-for-use databases maintain ‘teaching’ or ‘demo’ subsets of their data collections [*e.g.* the Cambridge Structural Database (500 structures; http://www.ccdc.cam.ac.uk/Solutions/FreeSoftware/Pages/CSDTeachingDatabase.aspx; accessed 2014–03–25) and the Inorganic Crystal Structure Database (3592 structures; http://icsd.ill.eu/icsd/index.html; accessed 2014–03–25)], these are typically small and do not permit demonstration of real-life problems to students. In contrast, open databases allow students to mine hundreds of thousands of records, teaching them how to handle the amounts of data relevant for contemporary crystallography. Moreover, since the software used to produce COD is also open source, students who are so inclined can participate in the development of different aspects of the database. Such early involvement and a ‘learning-by-doing’ approach results in publications co-authored by the students and gives useful themes for bachelor’s and master’s degree theses (Merkys, 2013 ▸). It is important to note that the public domain dedication of COD by its creators permits the full use of COD in any university or institution, and indeed COD has been widely used by such institutions.

### 4.2. Three-dimensional printing in teaching crystallography   

The enhanced economic activity that came with the expiration of fundamental patents on three-dimensional printing (3ders.org, 2014 ▸) and other recent developments led to the forecast that the market for three-dimensional printing services and materials will grow from USD 2.5 billion in 2014 to USD 10.8 billion in 2018 (Canalys, 2014 ▸). An ever growing hobbyist–maker movement–culture (The Economist Technology Quarterly, 2011 ▸; Bowyer, 2014 ▸) ‘has attracted the interest of educators concerned about students’ disengagement from STEM subjects in formal educational settings’ (Sharples *et al.*, 2013 ▸).

College educators around the world are now producing their own three-dimensional printed crystallographic models (Stone-Sundberg *et al.*, 2015 ▸). Their papers are often published in journals and the proceedings of conferences that are dedicated to college education. Many scientific libraries do not subscribe to these journals and article series with educational focus. We take, therefore, the opportunity here to review these efforts briefly for the benefit of the crystallographic community. We do this in the spirit of the online trade journal *Chemical and Engineering News* (Halford, 2014 ▸), which reported recently on the activities of three different groups of researchers/college educators that are working on the three-dimensional printing of crystallographic models.

Timothy Herman (Herman *et al.*, 2006 ▸), Arthur Olson (Olson *et al.*, 2007 ▸) and their respective co-workers pioneered the field more than ten years ago. A community of enthusiasts of three-dimensional printing of crystallographic models is currently forming around a dedicated web site/wiki that Vincent Scalfani created and maintains at the University of Alabama Science Libraries (Scalfani, 2014 ▸). On this site, there are links to relevant papers, open-access software and a listserv discussion group (3D Printing Crystallography Group, 2014 ▸), as well as to a total of eight research groups. Interested readers may visit this site (Scalfani, 2014 ▸) and contact Vincent by email (3DP-XTAL-request@LISTSERV.UA.EDU) to join the discussions.

Efforts are also underway to create open-access repositories for three-dimensional print files of small molecules (Scalfani *et al.*, 2014 ▸; Royal Society of Chemistry, 2014 ▸) and macromolecules (Hurt, 2014 ▸). More than 30 000 small-molecule CIFs from COD (Gražulis *et al.*, 2012 ▸) have so far been converted to three-dimensional print files at the Royal Society of Chemistry Crystal Data Repository (Scalfani *et al.*, 2014 ▸). The macromolecular three-dimensional print files of the NIH 3D Print Exchange (Hurt, 2014 ▸) were derived from the Worldwide Protein Data Bank (Berman *et al.*, 2003 ▸, 2007 ▸). The availability of open-access crystallographic data (Gražulis *et al.*, 2009 ▸, 2012 ▸) is, thus, leading to derived open-access data for the greater good. Links to these two derived databases of three-dimensional print files are also available on the above-mentioned web site/wiki (Scalfani, 2014 ▸).

**Figure 6:**
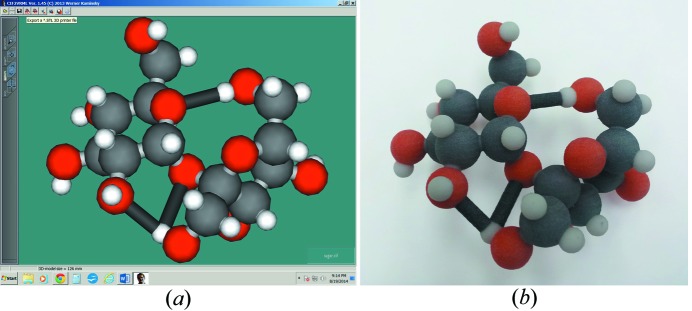
(*a*) Screenshot of the *Cif2VRML* program, which allows for saving as *.wrl (for color printing) and exporting as *.stl for printing on all kinds of inexpensive three-dimensional printers. (*b*) Color print of the sucrose molecule from a *.wrl file produced with *Cif2VRML*, whereby both heteropolar and hydrogen bonds are displayed.

For educators and researchers who would like to create their own three-dimensional print files, there is a ‘one click software solution’ (*Cif2VRML*; Kaminsky *et al.*, 2014 ▸) for the conversion from CIF to the three-dimensional printing file formats *.stl and *.wrl (see Fig. 6 ▸ *a*). While *.stl allows for monochrome printing using a variety of inexpensive three-dimensional printers and hobbyist machines, *.wrl allows for color printing (see Fig. 6 ▸ *b*). There are also Windows executable programs to produce three-dimensional print files for models of crystal morphologies (Kaminsky, 2005 ▸, 2007 ▸; Kaminsky *et al.*, 2014 ▸) and physical properties of crystals (Kaminsky, 2000 ▸; Kaminsky *et al.*, 2015 ▸). These programs are free to the individual college educator and require a license from the University of Washington at Seattle only if commercial usage is intended.

Three-dimensional printed models created by college educators can be excellent teaching tools because all models highlight certain features while unavoidably ignoring and misrepresenting other features (Charbonneau, 2013 ▸). A college educator may, therefore, choose to explain a complex concept with the help of a whole set of models that complement each other (and demonstrate at the same time the limitations of each individual model). One does not need to ‘own’ a three-dimensional printer in order to produce one’s own models. There are commercial (overnight) print shops which will do the actual printing (and necessary post-processing depending on the printing technique). (The three largest print shops are currently http://www.3dsystems.com/quickparts/, http://www.shapeways.com/ and http://www.sculpteo.com/en/.) What one has to provide, however, is the *.stl or *.wrl files of the models to be printed.

To our knowledge, there are so far one qualitative (Herman *et al.*, 2006 ▸) and two semi-qualitative (Höst *et al.*, 2013 ▸; Moeck *et al.*, 2014 ▸) assessments on the effectiveness of three-dimensional printed models in college education. All three of these studies subscribe to a constructivist philosophy of meaningful learning (Ausubel, 1968 ▸), where college students integrate new concepts encountered in the classroom or laboratory into their prior knowledge base. Both of the semi-quantitative studies (Höst *et al.*, 2013 ▸; Moeck *et al.*, 2014 ▸) were on small samples and are therefore not conclusive in a fully quantitative sense. For that, there are simply too many unknown parameters per participating student even if testing conditions could be assumed to be ideal. The goals of the two semi-qualitative assessments (Höst *et al.*, 2013 ▸; Moeck *et al.*, 2014 ▸) were to test the enhanced effectiveness of three-dimensional printed models over two-dimensional images from textbooks. The qualitative assessment (Herman *et al.*, 2006 ▸), on the other hand, was concerned with the enhanced effectiveness of three-dimensional printed models over computer-generated virtual reality pseudo-three-dimensional models.

**Figure 7:**
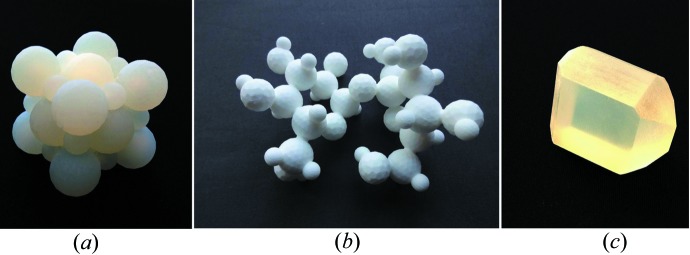
Three-dimensional printed models of (*a*) crystalline sodium chloride (table salt), (*b*) a sucrose (common sugar) molecule (without hydrogen bonds) and (*c*) a sucrose crystal. Models of these common household substances are utilized to explain concepts such as ionic, heteropolar, hydrogen and van der Waals bonding as well as crystals that contain either individual molecules (as in sugar) or cations and anions that are arranged in a charge balancing three-dimensional space lattice (as in table salt). (Reprinted with permission.)

Taking the pragmatic viewpoint of a crystallographer, one may point out that atomic arrangements and the world around us possess at least three spatial and one temporal dimension so that three-dimensional printed models should be more effective than two-dimensional images and computer-generated virtual reality pseudo-three-dimensional models. When three-dimensional models are a few centimetres in size so that they can be easily manipulated by hand, they stimulate both the visual and the tactile sense. One may consider dynamic processes as progressing in the additional dimension of time. As illustrated in two of the studies (Herman *et al.*, 2006 ▸; Höst *et al.*, 2013 ▸), this ‘fourth dimension’ can be included in ‘dynamical three-dimensional model kits’ that demonstrate time sequences of biological processes. Fittingly, these two studies deal with the effectiveness of three-dimensional structural biology models, which arguably need to be more dynamic than materials science models. Moeck *et al.* (2014 ▸), on the other hand, undertake an assessment of the effectiveness of three-dimensional printed materials science models (Figs. 7 ▸–9 ▸ ▸) in a 300 level introductory nanoscience and nanotechnology course without any science prerequisites. The captions of the figures give in each case a brief overview on the usage of the models in this course. Following Richard Feynman’s assertion that ‘Everything is made of atoms’ is the shortest sentence that conveys the most scientific meaning, a major goal of this course is to clarify that all of the complexity around the students is due to atoms forming materials with different physical, chemical and biological properties, which simply result from different atomic arrangements and types of bonds.

The photographs of the three-dimensional printed models in Figs. 7 ▸–9 ▸ ▸ were taken from the semi-quantitative study mentioned above (Moeck *et al.*, 2014 ▸). All of these models are several centimetres in size. In the ‘working for the greater good spirit’ of the forming community of enthusiasts of three-dimensional printing of crystallographic models (Scalfani, 2014 ▸; 3D Printing Crystallography Group, 2014 ▸), Portland State University’s Nano-Crystallography Group will make our current and future three-dimensional print files with educational relevance for materials science and engineering classes open access (Moeck, 2004 ▸).

## 5. Conclusions   

The methodologies, pedagogies, tips and tools presented here by no means represent a conclusive list of techniques for teaching crystallography to the world at large. We have attempted to compile a few descriptions of various in-class and extracurricular teaching models that have been proven successful. It is our wish as crystallographers and educators that our ideas may inspire others to incorporate the many fascinating concepts centered around the study of crystalline materials into their own courses. The International Year of Crystallography was proposed as a way to educate not only students already enrolled in STEM fields but everyone the world over. We hope that our experiences, chronicled here, may serve to further that aim.

## Supplementary Material

Appendix 1: Course outcomes for CH390. DOI: 10.1107/S1600576715016830/gj5144sup1.pdf

Appendix 2: Project scaffolding. DOI: 10.1107/S1600576715016830/gj5144sup2.pdf

Appendix 3. DOI: 10.1107/S1600576715016830/gj5144sup3.pdf

Appendix 4. DOI: 10.1107/S1600576715016830/gj5144sup4.pdf

## Figures and Tables

**Figure 4:**
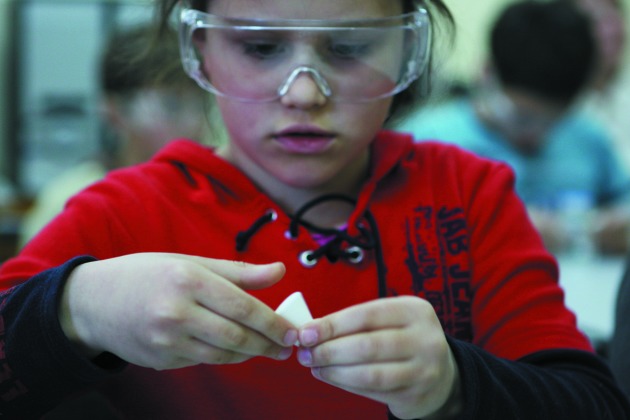
Children at work in the laboratory (photograph by Dr Anna Nartova; published with permission of the parents and the secondary school organizing the event.)

**Figure 5:**
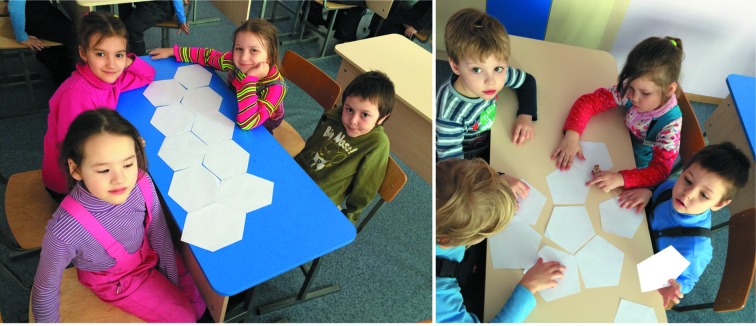
Kids at the children garden learning tiling in relation to periodic and aperiodic crystals (photograph by Sergey Arkhipov; published with permission of the parents and the secondary school organizing the event.)

**Figure 8:**
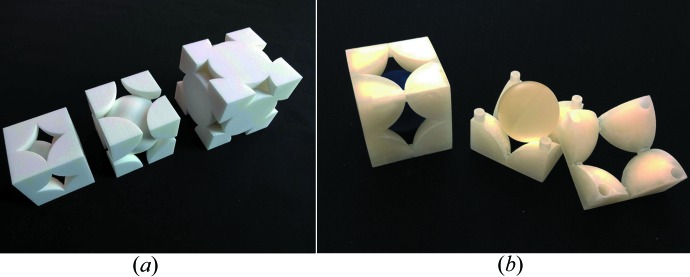
(*a*) Three-dimensional printed models of unit cells of the simple, body-centered and face-centered cubic packing of equal spheres. These models serve multiple purposes, *e.g.* to illustrate the unit cells of the three cubic Bravais lattices, the W and Cu structural prototypes, and the concept of different degrees of space filling in the hard sphere model. (*b*) Three-dimensional printed unit cell model of CsCl. Left: assembled pieces with a blue sphere in the middle. Middle and right: disassembled pieces and a translucent sphere, representing the Cs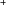 ion. Without these spheres in different colors, the two matching pieces represent when assembled the simple cubic Bravais lattice and simple cubic packing of equal spheres. This clarifies that the CsCl structural prototype possesses a simple cubic Bravais lattice and a basis consisting of two different atoms (rather than being ‘some kind of’ centered structure). (Reprinted with permission.)

**Figure 9:**
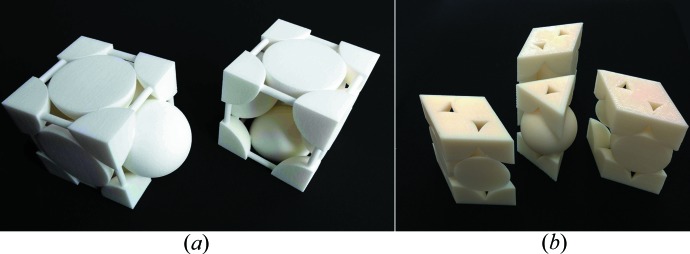
(*a*) Three-dimensional printed ‘opened-up’ version of the face-centered cubic unit cell with exposed tetrahedral and octahedral interstices (right) and complementary matching piece to ‘close’ the unit cell (left). (*b*) Three-dimensional printed ‘opened-up’ versions of the unit cell of the hexagonal densest packing of equal spheres with exposed octahedral interstices and a complementary matching piece to ‘close’ three unit cells. These models are used to demonstrate point and translation symmetries. These models are also utilized to derive other structural prototypes from the Cu and Mg structural prototypes by the filling of the various empty spaces with smaller spheres that represent other atoms. (Reprinted with permission.)

**Table 1:** Highlighted course goals for CH390

(*a*) Understand the organization of the library and know how to use library tools to obtain desired information and references
(*b*) Search for literature using appropriate queries for each database
(*c*) Demonstrate critical thinking by evaluating information, drawing conclusions from the literature and following a logical path of inquiry
(*d*) Understand and apply criteria for evaluating the authority and appropriateness of a document or information source
(*e*) Recognize the ethical component of complex situations

## Footnotes

1 Wilfrid Laurier Undergraduate Academic Calendar 2013/2014, Faculty of Science – Chemistry; http://legacy.wlu.ca/calendars/department.php?cal=1&d=1165&s=564&y=57.
