# Supplementary material for: Crystallographic education in the 21st century
Source: J Appl Crystallogr. 2015 Oct 13;48(Pt 6):1964–75. doi: 10.1107/S1600576715016830 (PMC4665665; doi:10.1107/S1600576715016830)
Supplement: Supplementary file 1 [file j-48-01964-sup1.pdf]

Crystallographic Education in the 21st Century

Saulius Gražulis   Amy Alexis Sarjeant   Peter Moeck
Jennifer Stone-Sundberg   Trevor J. Snyder
Werner Kaminsky   Allen G. Oliver   Charlotte L. Stern
Louise N. Dawe   Denis A. Rychkov   Evgeniy A. Losev
Elena Boldyreva   Joseph M. Tanski   Joel Bernstein
Wael M. Rabeh   Katherine A. Kantardjieff

September 4, 2015

Appendix 1

CH390 Learning Outcomes

Course outcomes for CH390 are from:

Information Competencies for Chemistry Undergraduates: the elements of information literacy. Special Libraries Association, Chemistry Division and American Chemical Society, Division of Chemical Information. 2nd ed. May 2011;

Upon completion of CH390, students should be able to:

1. Understand that science is filled with ethical judgments;

2. Recognize the ethical component of complex situations;

3. Analyze complex ethical problems and design appropriate solutions;

4. Understand the organization of the library and know how to use library tools (catalogs, databases, library web pages, subject guides, etc.) and library services (reserves, reference, interlibrary loan, etc.) to obtain desired information and references;

5. Understand the purpose and characteristics of different information-finding tools, e.g. catalogs, indexing and abstracting databases, subject guides, and web search engines, and choose appropriate tools for a particular information need;

6. Understand the content and organization of the print and online versions of a database;

7. Search for literature using searches as appropriate for each database, for example search by author, topic, chemical (name, CAS RN, structure, formula), and reaction;
8. Refine/limit literature searches (by topic, author, year, document type, language, etc);
9. Refine/limit substance/reaction searches (by structure, yield, steps, classification, etc.);
10. Understand what a cited/citing reference search is, why it is useful, and how to do it;
11. Request help from librarians, faculty, and teaching assistants when needed and consult online training materials when available;
12. Understand the nature and purpose of different types of scientific literature, including journals (communications, research articles, and review articles), magazines, patents, proceedings, dissertations, monographs, handbooks, encyclopedias and dictionaries, gray literature, and technical reports;
13. Be able to read and interpret citations for the different types of scientific literature;
14. Demonstrate the ability to cite using appropriate formatting and standard abbreviations;
15. Be familiar with software that allows for storing, managing, and formatting bibliographic references or citations;
16. Understand and apply criteria for evaluating the authority and appropriateness of a document or information source;
17. Demonstrate critical thinking by evaluating information, drawing conclusions from the literature, and following a logical path of inquiry;
18. Understand scientific ethics and accountability and have an awareness of intellectual property issues and developments in scholarly communications including those affecting author's rights, the use of copyrighted materials in research and instruction, and open-access initiatives related to the scientific literature;
19. Understand the general nature of the peer review process;
20. Be aware of the different methods for presenting research (articles, posters, oral presentations at scientific conferences, etc.);
21. Understand the reasons for citing the literature in one's own writing;
