# Supplementary material for: Crystallographic education in the 21st century
Source: J Appl Crystallogr. 2015 Oct 13;48(Pt 6):1964–75. doi: 10.1107/S1600576715016830 (PMC4665665; doi:10.1107/S1600576715016830)
Supplement: Supplementary file 2 [file j-48-01964-sup2.pdf]

Crystallographic Education in the 21st Century

Saulius Gražulis Amy Alexis Sarjeant Peter Moeck
Jennifer Stone-Sundberg Trevor J. Snyder
Werner Kaminsky Allen G. Oliver Charlotte L. Stern
Louise N. Dawe Denis A. Rychkov Evgeniy A. Losev
Elena Boldyreva Joseph M. Tanski Joel Bernstein
Wael M. Rabeh Katherine A. Kantardjieff

September 4, 2015

Appendix 2

CH390 Term Research Topics Winter 2014

There are four major term projects in CH390; an annotated bibliography, a review-style term paper, a poster and an oral presentation. You will focus all four of your term projects on a single topic. The United Nations has declared 2014 as the International Year of Crystallography (http://www.iycr2014.org/), and so your research topic will focus on some aspect of crystallography.

By Friday, January 10, you must e-mail your course instructor (ldawe@wlu.ca) with your top three selections from the following list. Also, include in your e-mail a short description of what you like in chemistry (ex. "I like math and physics" or "I like proteins" or "I like pharmaceutical drug design") so that I can ensure that even if you do not get your #1 pick, that you do get a subject related to your interests.

Note that many of these topics are very broad. You must focus your term research on the crystallographic aspects (for example, the role of X-ray crystallography in the determination of the structure and function of the ribosome.)

1. History of Small Molecule Crystallography in Canada;
2. History of Protein/Macromolecular Crystallography in Canada;
3. Multiscale models for complex chemical systems (2013 Nobel Prize – Chemistry);
4. G-protein-coupled receptors (2012 Nobel Prize – Chemistry);
5. Quasicrystals (2011 Nobel Prize – Chemistry);
6. Two-dimensional material graphene (2010 Nobel Prize – Physics);

7. Structure and function of the ribosome (2009 Nobel Prize – Chemistry);
8. Molecular basis of eukaryotic transcription (2006 Nobel Prize – Chemistry);
9. The fullerene form of carbon (1996 Nobel Prize – Chemistry);
10. Neutron Diffraction (1994 Nobel Prize – Physics);
11. Direct methods for the determination of crystal structures (1985 Nobel Prize – Chemistry);
12. Nanocrystals as Clathrates;
13. Crystals that exhibit large negative linear compressibility;
14. Highly Porous Materials for Clean Energy Applications;
15. Small Angle X-ray Scattering of Polymers;
16. Small Angle X-ray Scattering of Biological Macromolecules;
17. Endohedral Fullerenes;
18. Biomineralization;
19. Strong and Weak Hydrogen Bonding;
20. Computational and Crystallographic Studies of Transition Metal-Main Group Multiple Bonding;
21. Time-Resolved Biochemical Crystallography;
22. Pharmaceutical Polymorphism;
23. Developments in Inorganic Crystal Engineering;
24. High Pressure Crystallography;
25. Structure determination of molecular solids from powder X-ray diffraction data;
26. Magnetic Structure Determination;
27. Applications of the Cambridge Structural Database in Chemical Education;
28. Halogen bonding in supramolecular chemistry;
29. Phase Transitions in Inorganic Materials;
30. Innovations in Crystal Growing Techniques;
31. Dorothy Hodgkin;

32. Rosalind Franklin;
33. John Kendrew and Max Perutz;
34. Geoffrey Chang;
35. Synchrotron radiation sources – Diamond, CERN, Canadian Light Source and others;
36. Anomalous diffraction and the phase problem;
37. A topic of your own interest/suggestion;
