# Supplementary material for: Crystallographic education in the 21st century
Source: J Appl Crystallogr. 2015 Oct 13;48(Pt 6):1964–75. doi: 10.1107/S1600576715016830 (PMC4665665; doi:10.1107/S1600576715016830)
Supplement: Supplementary file 3 [file j-48-01964-sup3.pdf]

Crystallographic Education in the 21st Century

Saulius Gražulis Amy Alexis Sarjeant Peter Moeck
Jennifer Stone-Sundberg Trevor J. Snyder
Werner Kaminsky Allen G. Oliver Charlotte L. Stern
Louise N. Dawe Denis A. Rychkov Evgeniy A. Losev
Elena Boldyreva Joseph M. Tanski Joel Bernstein
Wael M. Rabeh Katherine A. Kantardjieff

September 7, 2015

Appendix 3

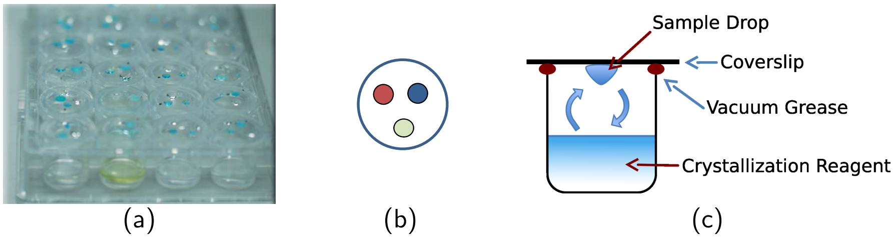

Figure 1: Micro-crystal Growth Setup. the hanging drop vapor diffusion method is used to grow micro size crystals. (a) A 24-well VDX crystallization plate from Hampton Research (Cat. HR3-306) is utilized to set up hanging drop vapor diffusion crystallization trials of the desired chemicals. The wells are individually sealed with 22 mm diameter circular plastic cover slides similar to those used in regular optical microscope. It is recommended to acquire plates with sealant for time and technical constrains. It will be technically challenging for the students to apply an even coat of the sealant on the individual wells. (b) Three different 4 μl drops of the chemical solution is placed on a cover slide that seals an individual well. All 24 wells will be sealed by cover slips that contains the same three chemical drops. The difference between the different wells are the crystallization reagents in the reservoirs. (c) 400 μl of the different crystallization reagents will be placed in the reservoirs of the 24-well plate. Different crystallization screens are available commercially.

At NYUAD, the crystal growth project consists of two parts that involve the crystal growth of macro and micro size crystals. For the macro-crystal growth, a saturated or supersaturated solution of the chemical is prepared using a magnetic stirrer, where a supersaturated concentration is reached upon heating the solution to ≈60 °C. For example, to get saturated copper sulfate

solution, dissolve 37.5 g of copper sulfate in 100 *ml* water. The solution then placed in different glassware including a beaker, flask, or test tube as well as in plastic container like Petri dishes or small jars. The containers with different materials, sizes, and shapes can affect the quality and size of the crystals. Also, the evaporation rate will affect the crystal quality by fully or partially closing the containers to limit the rate of evaporation. If multiple small size crystals are acquired, then a slower evaporation rate can promote the growth of large crystals.

For the second part of the project, the growth of micro-crystals, the hanging drop vapor diffusion method is used to grow micro size crystals similar to those used in protein crystallization, where a small quantity of the 50% saturated chemical solution is placed on a plastic cover slide that is placed on a well of a 24-well VDX crystallization plate from Hampton Research (Cat. HR3-306, Fig. 1a). Three different drops can be placed on the same cover slip to maximize the number of chemicals screened in the same plate (Fig. 1b) with extra precaution is needed not to mix the different drops by avoiding shaking or dropping the plate. The drops will equilibrate with different crystallization solutions placed in the reservoirs of the 24-well plate. Various crystallization screens are commercially available including a 100 different crystallization conditions from the Crystal Screen and Crystal Screen 2 available from Hampton Research (Cat. HR2-110 and HR2-112, respectively).

Crystallization experiments can be set up as follows: place 400 μl of crystallization solution into each reservoir of a VDX plate. Prepare 1 *ml* of 50% saturated inorganic chemical solution and place 4–5 μl drop of the chemical solution on 22 *mm* round cover slip. Cover the pre-greased well with the cover slip to seal the well and allow the crystallization solution to equilibrate with the drop. This process should be carried in short amount of time to prevent evaporation and the sample to dry.

The drops that contain a lower concentration of the crystallization agent will achieve a similar concentration to that in the reservoir by vapor diffusion. Water vapor leaves the drop and the concentration of the crystallization reagent increase until equilibration is reached when the concentration of the crystallization reagent in the drop is similar to that in the reservoir (Fig. 1c).
