# Supplementary material for: Crystallographic education in the 21st century
Source: J Appl Crystallogr. 2015 Oct 13;48(Pt 6):1964–75. doi: 10.1107/S1600576715016830 (PMC4665665; doi:10.1107/S1600576715016830)
Supplement: Supplementary file 4 [file j-48-01964-sup4.pdf]

Crystallographic Education in the 21st Century

Saulius Gražulis Amy Alexis Sarjeant Peter Moeck
Jennifer Stone-Sundberg Trevor J. Snyder
Werner Kaminsky Allen G. Oliver Charlotte L. Stern
Louise N. Dawe Denis A. Rychkov Evgeniy A. Losev
Elena Boldyreva Joseph M. Tanski Joel Bernstein
Wael M. Rabeh Katherine A. Kantardjieff

September 7, 2015

Appendix 4

Table 1: Amount of substances used in the experiment to grow crystals of pure copper

Substance	Amount, grams
Copper Sulfate	50
Sodium Chloride	150
Iron (e.g. steel pins)	17

Layers are prepared as shown in Fig. 1, flooded with saturated solution of sodium chloride (white salt from the shop) as a final step. System is stored till formation of copper crystals observed (approximately a week for 10 cm diameter beaker) or later to form more and bigger crystals. Saturated solution of sodium chloride was added every day to preserve the original level of solution. After a week crystals were removed manually from the original beaker to the new one and were washed with distilled water. The step by step procedure is shown at Fig. 2.

Crystals were dried in the air. Photos of final crystals are presented at Fig. 3. Long term storage was done using diluted sulfuric acid.

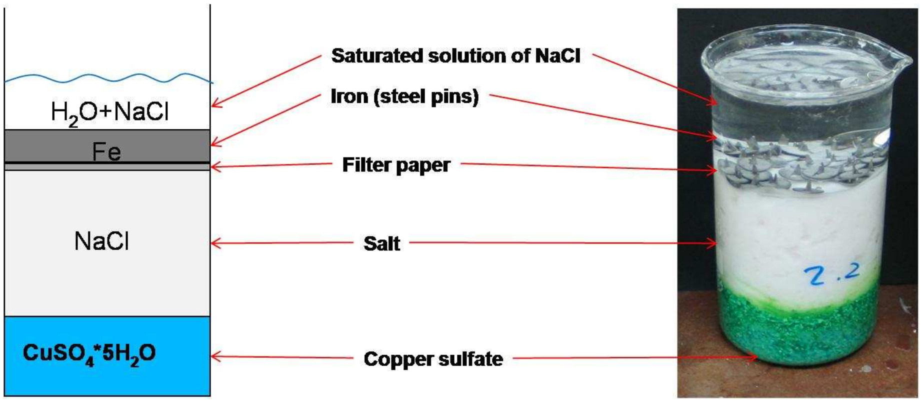

Figure 1: The scheme (left) and the photo (right) of the experimental setup. Consecutive layers of copper sulphate, sodium chloride, filter paper, iron source (steel pins at photo) flooded with saturated solution of sodium chloride.

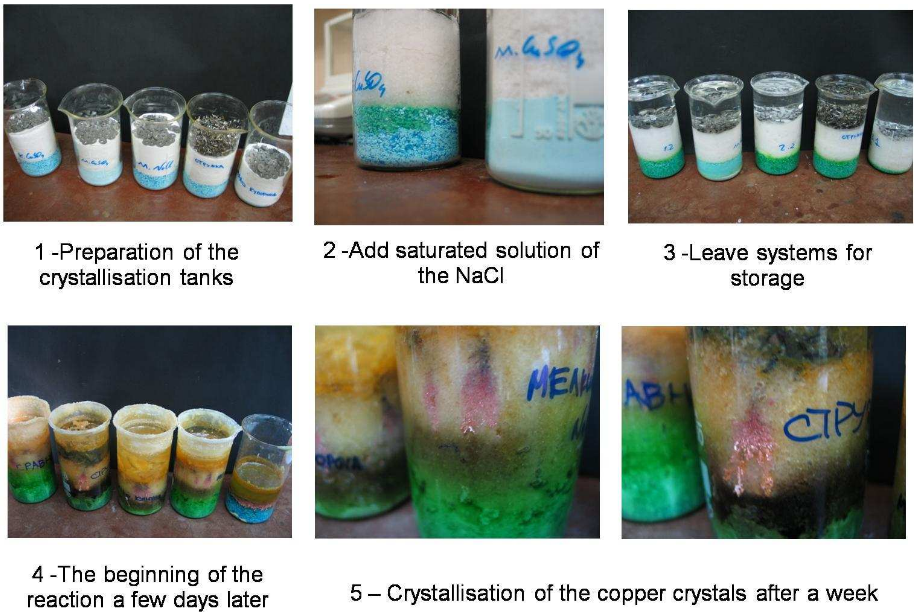

Figure 2: Step by step scheme of copper crystal growth.

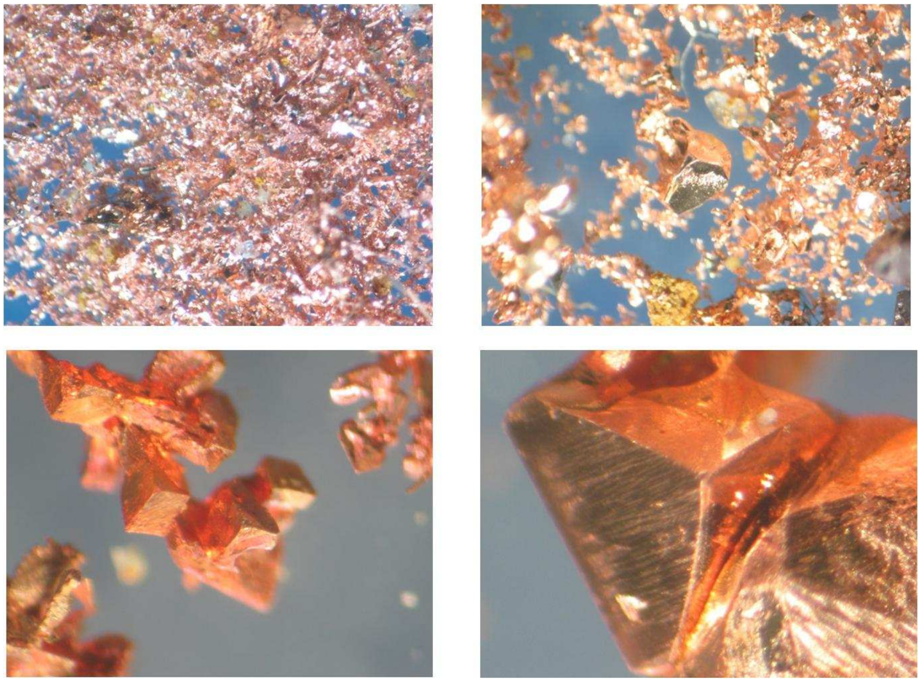

Figure 3: Photos of copper crystal under different microscope zoom.
